# *Carica papaya* L. Leaves: Deciphering Its Antioxidant Bioactives, Biological Activities, Innovative Products, and Safety Aspects

**DOI:** 10.1155/2022/2451733

**Published:** 2022-06-09

**Authors:** Anshu Sharma, Ruchi Sharma, Munisha Sharma, Manoj Kumar, Mrunal Deepak Barbhai, José M. Lorenzo, Somesh Sharma, Mahesh Kumar Samota, Maria Atanassova, Gianluca Caruso, Mo. Naushad, Radha, Deepak Chandran, Pramod Prakash, Muzaffar Hasan, Nadeem Rais, Abhijit Dey, Dipendra Kumar Mahato, Sangram Dhumal, Surinder Singh, Marisennayya Senapathy, Sureshkumar Rajalingam, Marthandan Visvanathan, Lejaniya Abdul Kalam Saleena, Mohamed Mekhemar

**Affiliations:** ^1^Department of FST, Dr. Yashwant Singh Parmar University of Horticulture and Forestry, Nauni, Solan 173230, India; ^2^School of Bioengineering and Food Technology, Shoolini University, Solan 173229, Himachal Pradesh, India; ^3^Sri Shankara Cancer Hospital and Research Centre, Bengaluru, Karnataka 560004, India; ^4^Chemical and Biochemical Processing Division, ICAR—Central Institute for Research on Cotton Technology, Mumbai 400019, India; ^5^Centro Tecnológico de la Carne de Galicia, Parque Tecnológico de Galicia, Avd. Galicia no. 4, San Cibrao das Viñas, 32900 Ourense, Spain; ^6^Área de Tecnología de los Alimentos, Facultad de Ciencias de Ourense, Universidad de Vigo, 32004 Ourense, Spain; ^7^Horticulture Crop Processing Division, ICAR-Central Institute of Post-Harvest Engineering and Technology, Abohar, Punjab, India; ^8^Chemical Engineering, UCTM, Sofia 1734, Bulgaria; ^9^Department of Agricultural Sciences, University of Naples Federico II, Portici, Naples, Italy; ^10^School of Biological and Environmental Sciences, Shoolini University of Biotechnology and Management Sciences, Solan 173229, India; ^11^Department of Veterinary Sciences and Animal Husbandry, Amrita School of Agricultural Sciences, Amrita Vishwa Vidyapeetham University, Coimbatore 642109, India; ^12^Agro Produce Processing Division, ICAR—Central Institute of Agricultural Engineering, Bhopal 462038, India; ^13^Department of Pharmacy, Bhagwant University, Ajmer 305004, India; ^14^Department of Life Sciences, Presidency University, 86/1 College Street, Kolkata 700073, India; ^15^CASS Food Research Centre, School of Exercise and Nutrition Sciences, Deakin University, Burwood 3125, Australia; ^16^Division of Horticulture, RCSM College of Agriculture, Kolhapur 416004, India; ^17^Dr. S. S. Bhatnagar University Institute of Chemical Engineering and Technology, Panjab University, Chandigarh 160014, India; ^18^Department of Rural Development and Agricultural Extension, College of Agriculture, Wolaita Sodo University, Wolaita Sodo, SNNPR, Ethiopia; ^19^Department of Agronomy, Amrita School of Agricultural Sciences, Amrita Vishwa Vidyapeetham Univer-sity, Coimbatore 642109, India; ^20^Department of Seed Science and Technology, Amrita School of Agricultural Sciences, Coimbatore, Tamil Nadu 642109, India; ^21^Department of Food Science and Nutrition, Faculty of Applied Sciences, UCSI University, 56000 Cheras, Willayah Persekutuan, Kuala Lampur, Malaysia; ^22^Clinic for Conservative Dentistry and Periodontology, School of Dental Medicine, Christian-Albrecht's University, 24105 Kiel, Germany

## Abstract

The prevalence of viral infections, cancer, and diabetes is increasing at an alarming rate around the world, and these diseases are now considered to be the most serious risks to human well-being in the modern period. There is a widespread practice in Asian countries of using papaya leaves (*C. papaya* L.) as herbal medicine, either alone or in combination with prescribed medications, to treat a variety of ailments. The importance of conducting the necessary descriptive studies in order to determine the safety of papaya leaf consumption is also emphasized in the context of their application in the healthcare sector. Electronic databases such as Google Scholar, Scopus, and PubMed were used to gather information on papaya leaves, their therapeutic potential, and clinical evidence-based studies. The literature was gathered from publications on papaya leaves, their therapeutic potential, and clinical evidence-based studies. The antidengue, anticancer, antidiabetic, neuroprotective, and anti-inflammatory effects of papaya leaves discussed in this article are supported by evidence from preclinical, *in vivo*, *in vitro*, and clinical trial studies, as well as from other sources. Leaves have been investigated for their mechanism of action as well as their potential to be used in the development of novel herbal products for the health business. According to the reports gathered, only a small number of research demonstrated that leaf extract at high concentrations was hazardous to certain organs. The collective literature reviewed in this review provides insights into the use of papaya leaves as a cure for epidemic diseases, highlighting the phytochemical composition and pharmacological attributes of papaya leaves, as well as the results of various preclinical and clinical studies that have been conducted so far on the subject. The review clearly demonstrates the successful medical evidence for the use of papaya leaf extracts in the healthcare system as a supplemental herbal medication in a variety of clinical settings.

## 1. Introduction

Chronic diseases are becoming an increasingly serious hazard to public health, necessitating the implementation of nutrition-based strategies to combat them. It can be difficult to obtain medical care for certain disorders, and the consumption of staple functional foods is vital in terms of preventing such ailments. Medical care accounts for 10 to 20% of the changeable contributors to human health, whereas social determinants, specifically healthy eating habits, account for 80 to 90% of the adjustable contributors. Healthy plant-based diets are more environmentally friendly and are connected with a lower risk of obesity, type 2 diabetes mellitus, viral infections, and some malignancies, among other health benefits. When it comes to addressing chronic human diseases, functional agriculture, specifically the cultivation of functional food crops like papaya leaves (*Carica papaya* L.), has emerged as a new frontier in nutritional research. The creation of functional food crops employing cutting-edge technology in combination with approaches from crop science, food science, and preventive medicine is therefore a significant topic of research [1].

*C. papaya* L. belongs to the family Caricaceae and is commonly known as papaya, pawpaw, and kates. It is a perennial horticultural shrub originated from Mesoamerican Centre, Central America, and southern Mexico [2–4] and is mainly cultivated in the tropical and subtropical regions of Brazil, Australia, Malaysia, China, India, Thailand, Myanmar, Philippines, and other adjoining [5]. Papaya is not only cultivated for the ripe sweet fruit, even other parts of the plant such as seeds, leaves, roots, flowers, barks, and latex have been traditionally used worldwide for the preparation of various medicinal formulations [6, 7]. However, leaves have been emerged as one of the most useful parts with plethora of health-promoting compounds and activities. In traditional medicines, the decoction of papaya fresh leaves is added into a tea to cure malaria, whereas dry and cured leaves are used as cigar for smoking by persons suffering from respiratory disorders such as asthma. Fresh young leaves of papaya are consumed as a leafy vegetable after steaming in some countries. In India, boiled leaves of papaya are recommended by Ayurveda practitioners as relief from malarial and dengue fevers as papaya leaf extract is considered effective to elevate platelet count and red and white blood cells in patients after suffering from viral fever [8]. The extract has also been known to protect the patients against the sickling of red blood cells [9]. In many parts of Asia, papaya leaves are used for the treatment of beriberi [10]. Papaya leaves have been identified to have more than fifty bioactive components and therefore useful in the treatment of different human diseases [11, 12]. Scientific studies revealed the existence of considerable levels of glycosides, flavonoids, alkaloids, saponins, phenolic compounds, amino acids, lipids, carbohydrates, enzymes, vitamins, and minerals in papaya leaves [13, 14]. The crude form of ethyl acetate isolates of papaya leaves has very good antiplasmodial effect against *Plasmodium falciparum* and *P. falciparum*-resistant strains [15, 16]. Although the leaves of papaya are used in Ayurvedic medicines, the juice from green leaves has been gaining the attention of today's consumers as a functional food due to its potent antiviral and immunity-enhancing properties [17]. Tea prepared from the juice extracted from papaya leaves is also used as a synergistic therapeutic dietary supplement for patients suffering from the oxidative stress-related diseases because of its strong antioxidant potential [18]. Few of the studies reported that fresh papaya leaves possess antiseptic properties, while the dried leaves can be used as a tonic to purify the blood and to improve digestion. Leaf juice of papaya is now being known for its potent anticancer [19], antioxidative [4, 5], anti-inflammatory [7], antimicrobial [20], and antisickling properties [21] along with nephron protective [22], hepatoprotective [23], hypoglycaemic, and hypolipidemic effects [24] against toxins in the human system. In fact, polar isolates of papaya have exhibited antihuman immunodeficiency virus (HIV), analgesic, and wound healing properties [25]. The imbalance in the activity of free radicals and the cellular antioxidant system is implicated to various lethal conditions such as cancer and cardiovascular diseases [26]. Recent research studies have been focusing on all-natural antioxidant-enriched plant parts, and in particular, papaya leaves are currently being consumed because a medical assessment of the leaf extract exhibited antiproliferative activity on cancerous cells along with its immune modulatory effects. There is as significant number of reviews regarding the functional properties of papaya fruits, but limited reviewing available relevant to the phytochemicals, biological activity, medical studies, and scope of using papaya's leaves in health industry. Therefore, this review is aimed at bridging this gap for better utilization of papaya leaves in the future due to their great medicinal potential. Electronic databases such as Google Scholar, Scopus, and PubMed were used to gather information on papaya leaves, their therapeutic potential, and clinical evidence-based studies. The literature was gathered from publications on papaya leaves, their therapeutic potential, and clinical evidence-based studies. The antidengue, anticancer, antidiabetic, neuroprotective, and anti-inflammatory effects of papaya leaves discussed in this article are supported by evidence from preclinical, *in vivo*, *in vitro*, and clinical trial studies, as well as from other sources. Leaves have been investigated for their mechanism of action as well as their potential to be used in the development of novel herbal products for the health business. The collective literature reviewed in this review provides insights into the use of papaya leaves as a cure for epidemic diseases, highlighting the phytochemical composition and pharmacological attributes of papaya leaves, as well as the results of various preclinical and clinical studies that have been conducted so far on the subject. The review clearly demonstrates the successful medical evidence for the use of papaya leaf extracts in the healthcare system as a supplemental herbal medication in a variety of clinical settings.

## 2. Phytochemical Composition of Papaya Leaves

**Figure 1:**
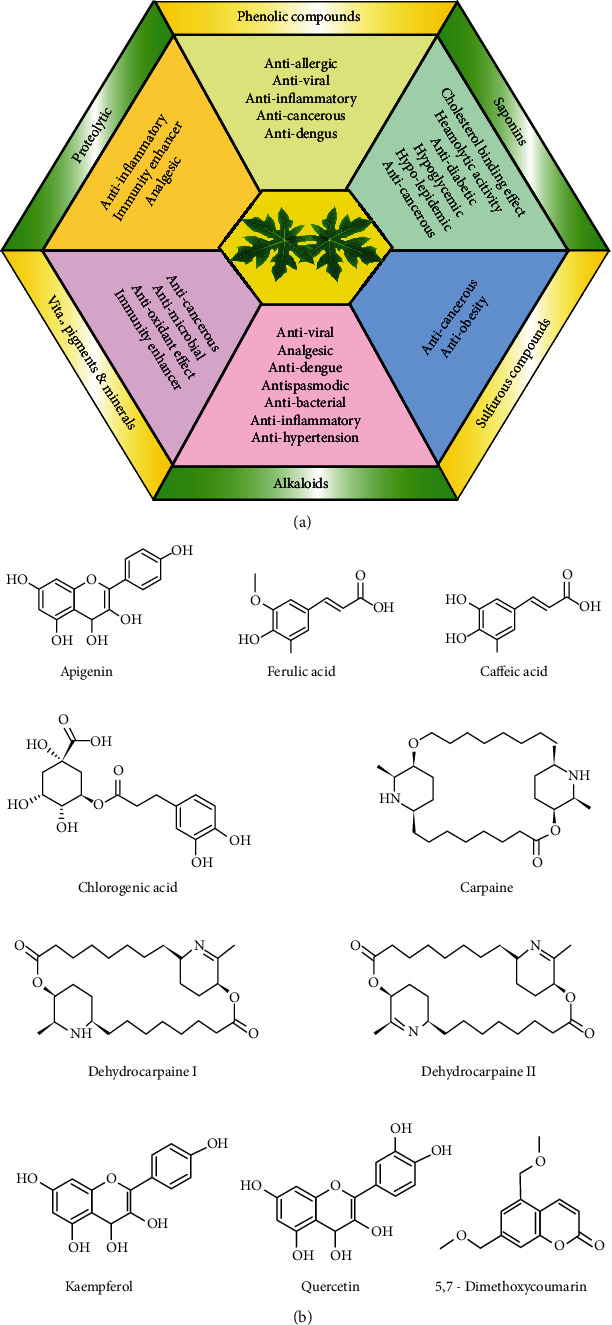
Chemical constituents and structure of some important compounds of *C. papaya* leaves. (a) Constituents of *C. papaya* leaves along with functional uses; (b) chemical structures of important bioactive compounds present in *C. papaya* leaves.

Phytochemicals are chemical components, naturally found in different parts of plants, which make many species beneficial for therapeutic uses. Indeed, leaves of papaya are known to have various health-promoting phytochemicals, as it arose from chemical analysis performed in various studies which clearly illustrated the presence of significant amounts of alkaloids, saponins, glycosides, flavonoids, phenolic compounds, enzymes, amino acids, lipids, carbohydrates, vitamins, and minerals [13]. There were seven flavonoids found in papaya leaves, which were named as quercetin, kaempferol 3-rutinoside, quercetin3-(2G-rhamnosylrutinoside), quercetin 3-rutinoside, kaempferol 3-(2G-rhamnosylrutinoside), myricetin 3-rhamnoside. Caffeic acid, protocatechuic acid, quercetin, 5,7-dimethy coumarin, p-coumaric acid, and chlorogenic acid are among the phenolic substances found in the leaves [19]. There is evidence to suggest that leaves contain a wide range of phytochemicals, including carpaine, kaempferol 3-(2G-glucosylrutinoside), kaempferol 3-(2^″^-rhamnosylgalactoside), 7-rhamnoside, kaempferol 3-rhamnosyl-(1->2)-galactoside-7-rhamnoside, luteolin 7-galactosyl-(1->6)-galactoside, orientin 7-O-rhamnoside, 11-hydroperoxy-12,13-epoxy-9-octadecenoic acid, palmiticamide, and 2-hexaprenyl-6-methoxyphenol [25]. Due to these potent bioactive components, extracts of the aforementioned leaves can be used to prepare nutraceuticals and herbal medicinal formulations. Chemical constituent and structure of some important compounds of *C. papaya* leaves are illustrated in Figure 1. There were reports that C. papaya leaves were used with other herbs to heal ailments. Traditional doctors in Nigeria use it to treat diabetes, while in Cameroon, they combine it with other herbs to treat malaria and other fungal infections and aboriginal Australians' record using decoctions of the leaf as an anticancer remedy [16, 19]. The functional bioactive components of leaves of papaya can elevate the overall antioxidant potential of blood. The leaves of papaya plant are well known to have papain, cystatin, chymopapain, tocopherol, phenolic acids, cyanogenic glucosides, glucosinolates, and vitamin C as main phytochemicals [27]. Mainly alkaloids, saponins, glycosides, phenolic compounds, and flavonoids are responsible for the anti-inflammatory and anticancerous properties of papaya leaves [28]. Vitamins, minerals, and amino acids of papaya leaves are quite helpful to improve the total haemoglobin, proteins, and immunity of human system [29]. Carpaine along with dehydrocarpaine I and dehydrocarpaine II are most important health-promoting and major bioactive components found in the leaves of papaya. Due to the presence of carpaine, these herbal leaves are utilized in Ayurveda formulations for treating various physical disorders and viral fevers such as dengue and chikungunya. The aforementioned alkaloid has the ability to calm high blood pressure and fast heart rate and is effective for the uterus marked relaxation, the bronchioles dilatation, and movement of the intestinal strips along with antiplasmodial properties [30]. Carpaine has also been reported to have potent anticancerous and antihelminthic properties [31]. Its concentration has been reported the highest in mature leaves of papaya, i.e., 9.30 mg/g, followed by fruit pulp, i.e., 4.90 mg/100 g, fruit peel, i.e., 1.99 mg/100 g, and seeds, i.e., 0.65 g/g [32]. Leaves constitute different components in varying proportions such as 8.3% of carbohydrates, 38.6% of vitamin C, 5.6% of pro- and 0.23% of phosphoric acid. A good amount of tannin (0.85 ± 10^−3^ ± 1.76 ± 10^−4^ M, 0.824%) in papaya leaf extract has been reported by a few researchers [33]. Papaya leaves has been found to have highest total phenolic compounds as 424.89 ± 0.22 mg GAE/100 g of the dry sample followed by the 339.91 ± 9.40 in unripe papaya, 272.66 ± 1.53 in ripe papaya, and 30.32 ± 6.90 mg GAE/100 g in seeds [34]. Due to the aforementioned bioactive compounds, a very good antioxidant potential of 90% has been recorded in its leaves already. Researchers also reported a good concentration of calcium and magnesium, i.e., 3480 mg/kg and 5928 mg/kg, respectively; other minerals like iron (558 mg/kg), zinc (33.4 mg/kg), manganese (22.88 mg/kg), chromium (7.50 mg/hg), and copper (2.16 mg/kg) were also found in fair amount [16]. Papaya leaves have shown the highest ascorbic acid content with the concentration of 85.6 mg/100 g followed by 45.8 mg/100 g in ripe papaya, 37.8 mg/100 g in unripe papaya, and 14.4 mg/100 g in its seeds [34]. Biological enzymes, viz., papain and chymopapain, are in abundance in the leaves of papaya [35]. The concentration of papain in papaya leaf extract varies from 0.054 to 0.002 mg/mL [35] and due to which very powerful digestive action even higher than pepsin is seen, important phytochemical constituents of leaves along with their functional properties and structures.

## 3. Bioactivities of Papaya Leaf Extract

Papaya leaves have a very long history in terms of its medicinal uses and have been utilized in many Asian countries for treating various ailments. Because of the presence of the aforementioned important functional constituents, they are used to cure corns, warts, constipation, weakness, amenorrhea, menstruation problems, eczema, sinuses, cutaneous tubercle, glandular tumour, diabetes, ulcers, hypertension, dengue, etc. [8]. Traditionally, Australian aboriginal people consume papaya leaf extract for its anticancerous activity [36, 37]. In addition to their various cancer-fighting components, *C. papaya* leaves contain a significant amount of nutrients to improve the immunity. Beside vitamins E, A, and C, they have vitamin B-17 whose concentrated form is used to cure cancer patients in conventional chemotherapy treatment. Phytochemicals of papaya leaves have been reported to prevent bone marrow depletion and platelet destruction [2]. Juice of papaya leaf is quite helpful to elevate platelet count and red blood and white blood cells to normalize blood clotting and to repair the liver [18].

### 3.1. Antioxidant Effect

Many phytonutrients found in plants, such as fruits and vegetables, have come to the attention of food experts and the general public in recent years for their potential health benefits. Due to concerns about synthetic antioxidants' toxicity, these phytochemicals are commonly marketed as natural antioxidants as an alternative. Oxidative compounds present in many plants have antibacterial, antiviral, and cancer-fighting properties. They also have an array of other health benefits [19, 38]. Papaya peels are discarded after consuming the fruit. However, they contain antioxidants. Oxidative damage caused by free radicals has major implications in many chronic diseases [25]. By inhibiting the creation of free radicals, antioxidants can aid our health. New sources of natural antioxidants that are both safe and economically viable are now being investigated. Researchers made silver nanoparticles from *C. papaya* peel extract (CPPE) and examined their antioxidative properties to see if they worked. We found that the concentration-dependent activity of AgNPs was 56% for synthesised AgNPs and 38% for commercially available CPPE [39]. According to a recent study, the antioxidant activity of methanolic extract of papaya leaf was assessed by measuring its ability to neutralise free radicals (DPPH) [40]. DPPH free radical scavenging capacity was found to be best in hexane extract and lowest in aqueous extract in another investigation using papaya seed extracts and the results showed [41]. Papaya leaf antioxidant activity has been studied by Nisa et al. using various cultivars, maturities, and solvents. During extraction, the solvents were water, methanol, and 70% ethanol. Results showed that water-extracted mature leaves had the highest antioxidant activity of any of the other types of leaves tested. PaMsrB1 (plant methionine sulfoxide reductase B1) from papaya leaf was studied with *Escherichia coli*, which has MBP (maltose-binding protein) at its N terminal protease activity, which assists in the digestion of MBP-tag and leads to the separation of the recombinant PaMsrB1. In the presence of dithiothreitol, the purified recombinant protein PaMsrB1 demonstrated reductase activity against methionine sulfoxide (MetSO). An affinity chromatography and LC/MS/MS study discovered several proteins that interact with PaMsrB1. Understanding the defensive mechanisms of PaMsrB1 against antioxidative stress is facilitated by these findings [41]. Antioxidant activity and total phenolic content (TPC) were measured by Ang et al. to determine the antioxidative capability of *C. papaya* peels. Ferric reducing/antioxidant power (FRAP) and the ABTS radical cation inhibition activity (ABTS-RCI) were used to evaluate antioxidant activities, and the Folin-Ciocalteu method was used to measure TPC. The TPC of the papaya peel was 15.18 g GAE/mL when extracted with 90% acetone (*v*/*v*) for 60 minutes. DPPH, FRAP, and ABTS assays found antioxidant activity of 37.34%, 19.70 *μ*g TE/mL extract, and 28.30%, respectively. The antioxidant potential of papaya peel may contribute to production of functional foods and nutraceutical in the near future utilising these papaya wastes [38]. Calvache et al. treated papaya peel residues with ethanol and drying them in a microwave oven to generate dietary fibre concentrates (DFCs), in order to demonstrate its antioxidant activity. Carotenoids, phenolics, ascorbic acid, proteocatechuic acid, manghaslin, quercetin 3-O-rutinoside, caffeoyl hexoside, ferulic acid, lutein, zeaxanthin, and beta-carotene were detected in the chromatographic analysis of the samples. Upon analysis of digestibility, it was found that about 65% of the polyphenols associated to peel DFCs were potentially bioaccessible in the small intestine and that the portion of indigestible fiber had antioxidant capacity [42]. *In vitro* antioxidant activity of papaya peel extracts, and their effects on endogenous glutathione, superoxide dismutase, catalase, cyclo-oxygenase-2 (COX-2), cyclo-oxygenase-3, and DNA fragmentation in HepG2 cells were investigated by Salla et al. Papaya peel extracts contained significant amounts of gallic acid (18.06 *μ*g/g), caffeic acid (29.28 *μ*g/g), p-coumaric acid (38.16 *μ*g/g), ferulic acid (95.46 *μ*g/g), and quercetin (3.17 *μ*g/g). *In vitro* antioxidant capacity of papaya peels was determined by FRAP (31.86 *μ*M Fe^+2^/g), trolox equivalent antioxidant capacity (14.56 mM trolox equivalents/g), oxygen radical scavenging activity (30.88 mM TE/g), and 2,2-diphenyl-1-picrylhydrazyl radical scavenging ability (IC50 = 8.33 mg/mL). SOD, CAT, GPx, GR activity, and GSH levels were decreased by 3.1, 1.46, 2.87, 1.34, and 1.32 times, respectively, when oxidative stress was induced. *Papaya* peel extracts, on the other hand, significantly increased SOD, CAT, GPx, GR, and GSH activities in cells compared to cells exposed to oxidative stress. It was found that papaya peel extracts caused cell death by apoptosis cells by significantly reducing COX-2 activity, increasing caspase-3 activity, and triggering DNA fragmentation. Anticancer activities of papaya peel extracts may be attributed to the synergistic action of free radical scavenging, stimulation of antioxidant enzymes, and triggering apoptosis [43]. Similarly, antioxidant properties are directly and indirectly contributing towards imparting other bioactivities such as immunomodulatory activities, antiviral, antidiabetic, and others discussed in following subsections.  Table 1 provides an overview of *C. papaya*'s antioxidant properties.

### 3.2. Antiviral (Antidengue) and Antithrombocytopenic Effect

Dengue is an arboviral disease caused by dengue virus of the Flaviviridae family. Dengue fever occurs due to the infection transmitted by infected *Aedes aegypti* mosquito as a carrier of this virus [48]. The occurrence of this disease has increased by almost 30-fold in the previous three decades especially in developing countries. A number of infections caused by dengue virus ranges from 50 to 100 million per year [48], and every year, there is a new outbreak of dengue being reported. This viral infection leads to thrombocytopenia condition in infected patients [49]. The most common reason for thrombocytopenia is the poor production of platelets by the bone marrow, minimal survival of platelets, and sequestration of the platelets by the leptospirosis, malaria, dengue, and other viral infections. Major quantitative or qualitative dysfunction and reduction in the platelet count is the cause of mucocutaneous bleeding in the patients [50]. The platelet count drops below the normal level to an extent depending upon severity of viral infections. Moreover, viral fever is generally a self-limited illness which requires supportive care for complete recovery. Aspirin, antibiotics, nonsteroidal anti-inflammatory drugs, and corticosteroids must not be consumed by the patient as they are not so beneficial in viral infections. In fact, their consumption can cause gastritis or in severe cases internal bleeding too. One of the most disturbing aspects of the viral infections is that there are no effective antiviral agents available to treat their complications. *In vivo* studies have indicated quite beneficial effects of papaya leaf extract to improve immunity against infections and to increase platelet counts in thrombocytopenic patients after suffering viral infections [51].

**Figure 2:**
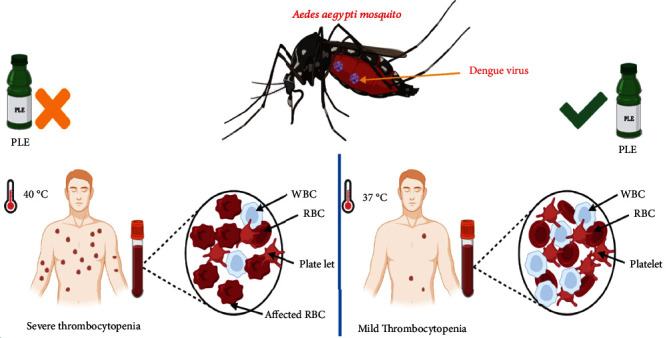
Antiviral (antidengue) and antithrombocytopenic effect of papaya leaves.

Various studies both with animal and human models have been conducted by researchers worldwide to confirm the anti-inflammatory effect [7] and platelet count improvement after administration of simple papaya leaf extract or ethanolic aqueous extract [8, 52–54]. The use of papaya extract is recommended to get early recovery in case of dengue with low platelet and red and white blood cell count [54]. As per few case studies conducted in recent years, its positive effect on total plate count is clearly demonstrated. Researchers orally administered a 25 mL papaya leaf extract to the dengue patients daily in the morning as well as evening times for five days continuously [17]. There was significant improvement in platelet count and white blood cells and neutrophils (NEUT) just after the second day of oral consumption, and the count reached their healthy normal level at the end of course. Research was conducted, which is the study of multiple platelet transfusions to a baby suffering from congenital thrombocytopenia. The patient did not respond well to phototherapy, intravenous immunoglobulin, and two exchange transfusions with antifungal therapy and antibiotics. However, papaya leaf extract oral administration as much as 20 mg/kg/dose of patient body weight, three times a day, exhibited quite a positive effect on platelet count without any side effects in the baby even during the follow-up period [55]. Antiviral (antidengue) and antithrombocytopenic effect of papaya leaf extract is shown in Figure 2.

Like these aforementioned studies, there are various preclinical and clinical studies confirming the therapeutic effect of papaya leaves on thrombocyte animal models and are summarized in Table 2 for further enlightenment on its therapeutic potential against thrombocytopenia in dengue infection. The action mechanism of papaya leaf extract shows very good stabilizing properties to prevent platelet lysis and inhibits heat-induced and hypotonicity-induced haemolysis of erythrocytes even at the lower extract concentration. In the latter respect, the extracts are likely to possess membrane-stabilizing attributes and protect blood cells against stress-induced destruction. This property might be useful in patients with dengue where papaya leaf extracts could prevent platelet lysis, due to the presence of functional phytochemicals [56]. Some studies reported that papaya leaf extracts increase the arachidonate 12-lipoxygenase, 12S type activity, and platelet-activating factor receptor significantly in the body which consequently increases the platelet production in the patients administered with papaya leaf extract. The flavonoids present in this extract have also been found efficient in suppressing a protease found in viral assembly [57]. Further, Sharma et al. reported that papaya leaf extract was able to significantly increase the platelet count in thrombocytopenic rats. The authors also confirmed *in vitro* antiviral activity of papaya leaf extract using dengue-infected THP-1 cells (human leukemia monocytic cell line), and the possible mechanism noted was reduction in both envelop protein and NS1 protein expression. Further in thrombocytopenic rats treated with papaya leaf extract, decreased erythrocyte damage was observed along with increase in IFN-*α* expression and thrombopoietin levels indicating its potential to be used as therapeutic that can help in improving the platelet count and exhibit antiviral agent against dengue fever [58].

### 3.3. Anticancer Activity

Cancer is a huge group of diseases which can affect any organ of the human body with abnormal body cell growth. Cancer is also commonly known by the name of malignant tumour, and the cells affected by this disease have a tendency to spread from the originating organ to others very rapidly. Nowadays, cancer is one of the major causes of death worldwide, with 9.6 million estimated deaths due to this lethal disease in a year [67]. Prostate, lung, colorectal, liver, and stomach cancers are commonly found in males, while breast, colorectal, thyroid, lung, and cervical cancers are the most reported in females [68]. The burden of this disease is continuously growing in the world, exerting tremendous emotional and financial strain on patients, their families and health systems, especially in low- and middle-income countries. Alternative therapy includes different plant extracts and their bioactive ingredients responsible for tremendous health improvement, including the prevention and treatment of cancer in many countries [69].

In one of the patents filed, it was declared that proliferation of cancer cells reduced while health improvement was noted when people having cancer (lung, stomach, colon, pancreatic, liver, neuroblastoma, ovarian, breast, solid, and blood cancer) were treated with brewed extract of papaya leaf or fractioned components [70]. Researchers also found ethanolic papaya leaf extracts with high levels of saponins more beneficial in suppressing cancer cell lines than aqueous extracts [71]. However, although there are significant sources denoting the anticancerous effects of papaya leaves, only a few studies have identified their exact effect on cancerous cells and mechanism of action [72].

Medicinal value of herbs is dependent on the chemical constituents present in them which are known for their positive pharmacological and physiological activities inside human system. Research studies on papaya has clearly denoted that the whole plant has great number of secondary metabolites [73] and are directly linked with the potent anticancerous activities inside human body [74, 75]. Recent studies were conducted to evaluate the effect of capsules of papaya on cancer-affected patients split in different age groups (pediatric: 3-8 years and adult: 18-72), including males and females aged, with different body weights and ethnic backgrounds. They noticed a significant decrease in cancerous growth of the patients treated with papaya leaf extract of 0.16 g/kg body weight compared to control. Their findings suggested that papaya leaf extract has a great effect as anticancerous therapy for prevention and cure of prostate cancer due to presence of the phytochemicals (amino acids, flavonoids, alkaloids, and phenolics). However, the authors suggested that thorough research and understanding, the mechanism of action as anticancer agent is required before promoting use of papaya leaf extract as adjuvant treatment of cancer [37]. There are *in vitro* studies which clearly indicated a significant positive effect of this herbal extract on various tumour cell lines. However, still, further research needs to be conducted to provide concrete evidence and mechanism of action of papaya leaf extract as anticancer agent.  Table 3 summarizes the results of studies conducted by various researchers to find out the medicinal potential of papaya against different kind of cancer cells.

**Figure 3:**
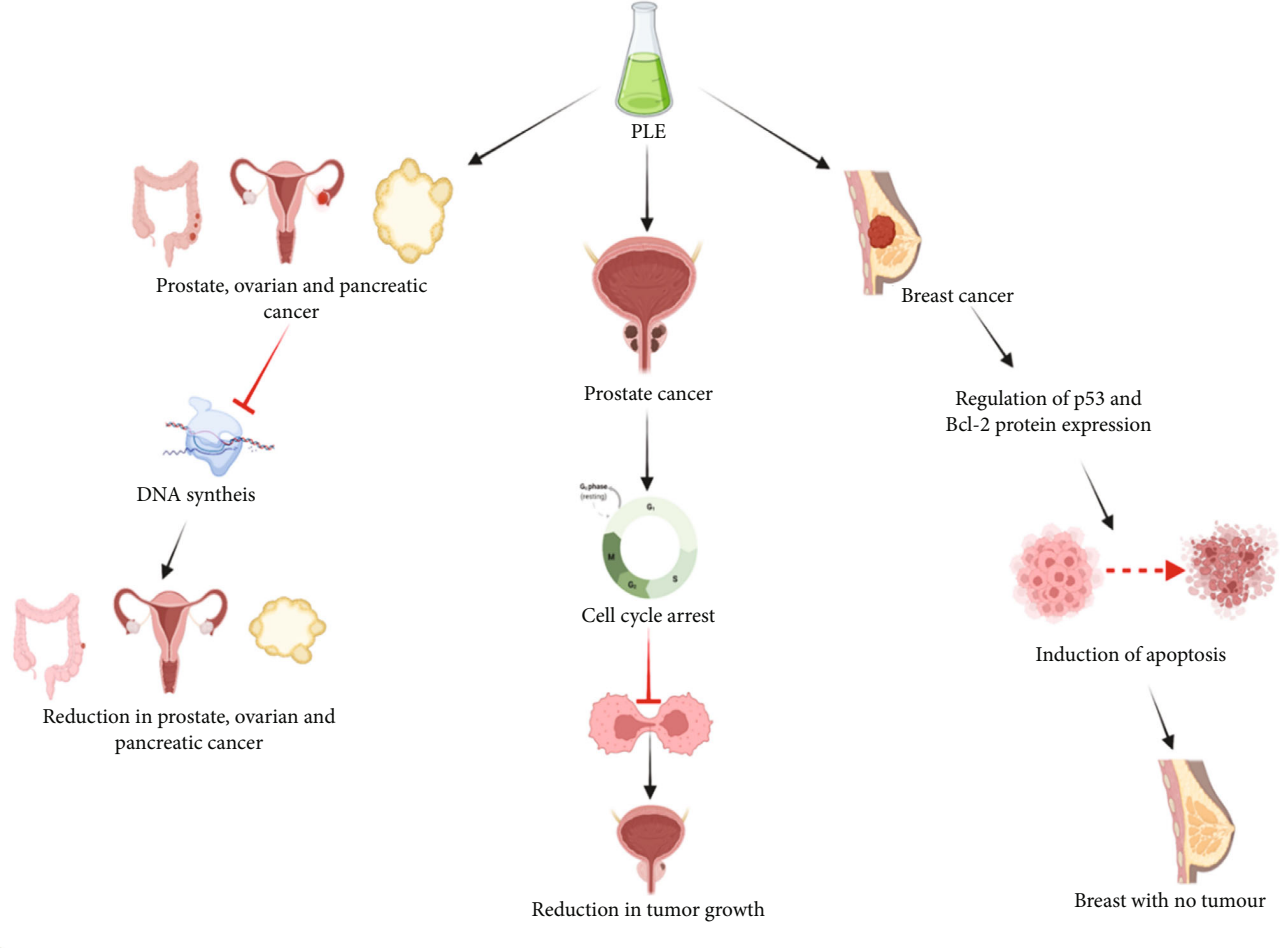
Anticancer activities of papaya leaf extract.

Papaya leaf extract (PLE) has ability to interact with a huge range of molecular targets and exerts disease preventive activities. The major molecular targets included in the anticancer prevention are inhibition of the activity of DNA topoisomerase I/II and change of signaling pathways. Previous studies suggested that anticancerous properties of papaya leaves might be due to two reasons, i.e., caspase-3/7 process activation and activation of p53-dependent mitochondrial pathway [36]. However, some researchers also revealed that PLE seizes the PCa cell in S phase of cell division, which leads to the cell death, thus responsible for anticancerous activity [79]. Molecular signaling pathways and their cross-talk plays an important role in imparting the anticancerous activity such as repression of DNA topoisomerase I/II activities, lower gene expression of Bcl-2, CDK 4, cyclin D1, and B1, PCNA and higher expression of genes like Bax, Bak, cleaved caspase 3, and P53 [36, 37]. Anticancer activity of papaya leaf extracts and possible mechanism of action is shown in Figure 3.

Papaya leaf extract's immune modulatory potential is responsible for increasing the concentration of nitric oxide, CD80, TNF-alpha, and various other interleukins (IL-12p70, IL-12p40) and induction of apoptosis in cancer cells [37, 69]. However, papaya leaf extract at concentrations of 5 Ig/mL on the release of cytokines exhibited percent inhibition of TNF-*α* (10.8%), IL-1*α* (12.5%), IL-1*β* (27.4%), IL-6 (42.9%), and IL-8 (8.4%) reported in some studies [80]. Possible mechanism of papaya leaf extract to act as an anticancerous agent is to lower down metastatic cancer by decreasing the extracellular matrix concentration which further acts as a chemo-attractants of PC-3 cells for adhesion as well as migration [76]. Thus, the extract shows the potential to reduce the procreation of cancer cells and conquer the process of DNA synthesis [70]. It might be inferred a prominent correlation between secretion of Th1 type cytokines and increased cancer cell toxicity, which may result in the antitumour activity of papaya leaf extracts [36].

### 3.4. Immunomodulatory Effects

Other studies showed the immunomodulatory potential of papaya leaf extract and the cytokine ELISA profile of PBMC and revealed that papaya leaf extract downregulates IL-4 and IL-2 excretion in supernatants of cultures in a dose-reliant manner and presumed that leaf extract of papaya may bring apoptosis in PBMC, like similar effect on cancerous cells [36]. However, secretion of Th1 type cytokines like IL-12p70, IL-12p40, TNF-*α*, or IFN-*γ* applicable to anticancer immunity was interestingly upregulated even at low concentrations of leaf extract, with minor effect on IL-15, IL-6, IL-5, and IL-10 production 9 [37]. Th1 (IFN-*γ*+ CD4+) vs. Th2 (IL-4+ CD4+) T cells are important mediators of inflammatory reactions, and they may be influenced by regulatory T cells [81]. Flow cytometry was used by Abdullah et al. (2011) to investigate the effects of *C. papaya* on cells from healthy people. Significant downregulation of IFN-*γ*+ CD4+ T cells, upregulation of IL-4+ CD4+ T cells, and upregulation of CD3+ CD4+ CD25+ CD127- T cells were observed after papaya consumption. Regulatory T cells were upregulated in male participants' *in vitro* cultures, and this was significantly associated with levels of IL-1 in culture supernatants [82]. There is a possibility that leaf extract of papaya may promote the control of Th2-mediated allergic ailment, or as an adjuvant of various vaccines by promoting an alteration from Th2 to Th1 type immune response [6]. The methanol (MeOH) extracts of *C. papaya* and on mice for 3 weeks were able to decrease the level of proinflammatory cytokine, and it was also found that use of standardized CPL aqueous extract (SCPLE) was significantly increasing the thrombocytes and phagocytic index in thrombocytopenic rats [80]. These findings help the researchers to screen out the anticancerous effects of papaya leaves on cancer cells *in vivo* studies. Studies conducted by various researchers with regard to immunomodulatory potential of papaya leaf extracts are summarized in Table 4.

### 3.5. Hypoglycemic and Antidiabetic Effects

Diabetes mellitus is a worldwide known disease caused by the failure of the pancreas to generate insulin or dysfunction of the human system to use insulin properly and has been emerging very rapidly worldwide. The increasing number of diabetes associated with the rough toxic effects of allopathic medicine has gained the attention of researchers to find out alternatives with minor or no side effects [83]. This is a serious long-term condition which has been considered one of the major reasons of deaths in adult group globally, with four million estimated deaths in recent years [81, 84]. The term diabetes mellitus itself shows many diseases of abnormal carbohydrate metabolism which is associated to hyperglycaemia. It is connected to impairment in the insulin secretion along with varying degrees of resistance against insulin action. Diabetes also increases the risk of other diseases and disorders such as obesity, ageing, heredity, and genetic mutilation of beta-cell function/insulin receptor. Many plants are known for their effective antidiabetic, phytochemicals in conventional and today's medicine as well [84, 85]. Even various literature reports have shown the positive effects of different plant parts for treating this disease [86].

The decreasing trend in the blood glucose level of the treated animals with the consumption of plant part extracts has revealed in many studies that the extract of plant portions possesses potent antidiabetic effects and can be utilized for its cure. Among different plant parts, papaya leaves have been used in traditional Ayurveda medicines for diabetes [84].

**Figure 4:**
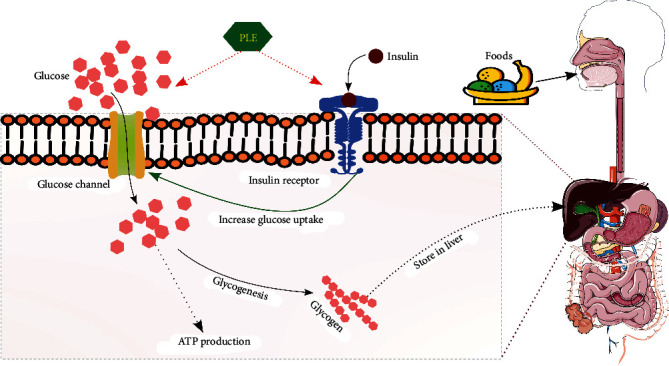
Antidiabetic activities of *Carica papaya* extract (CPE).

Preclinical studies available in literature shows the antidiabetic effect of papaya leaves on diabetic rats, but no investigation has been undertaken as a clinical trial on human beings to examine the antidiabetic effect of this herbal leaf extracts till date. Researchers suggest that papaya leaves could be alternative medicine in the treatment of diabetes as it has no side effects. The presence of significant number of phytochemicals of this leaf extract has a great effect in reducing other secondary complications raised by diabetes [87]. First preclinical study on therapeutic effects of papaya on diabetic Wistar rats was conducted in 2007. Papaya ethanolic leaf extract (5.0 mg/kg BW of male Wistar rats) for twenty-four hours was administered during the studies. A significant reduction in blood glucose of diabetic rats from 12.75 to 1.23 mmol/L within 24 hours of oral administration was observed by the researchers [88]. Studies conducted by various researchers are summarized in Table 5 and possible mechanism of papaya leaf extract as antidiabetic agent is shown in Figure 4.

Some studies suggested that the mechanism of action of aqueous papaya leaf extract consists in stimulating the beta cells with a higher pancreatic release of insulin, thus increasing peripheral glucose uptake or islets of Langerhans. Furthermore, reduced glycemic effect of papaya leaf extract is due to hampering of synthesis of fatty acids and cholesterogenesis decrease and due to an increasing amount of the latter parameters further increases the risk of overweight and diabetes. Different reports have shown that in diabetes, the islets appear to be preferentially affected by the destruction of insulin-secreting *β*-cells [95]. The mechanism triggered by papaya leaves consist in diminishing the lipid and carbohydrate hydrolyzing enzyme activity in the small intestines, which reduces disaccharides and triglycerides conversion into simpler easily absorbable monosaccharide and free fatty acids [96]. With the current available preclinical inputs, it is necessary to conduct more systematic, thorough cell line or animal model studies to prove the beneficial effect of papaya leaf extracts as hypoglycemic agent and before its implication as antidiabetic component.

### 3.6. Other Bifunctionalities of Papaya Leaves

No medical way has been proven to stop the death of brain cells in Alzheimer disease, though a few treatments only can help with both behavioural and cognitive symptoms. Aluminum leads to mitochondrial dysfunction with the generation of excessive free radicals and eventual damage in genetic material, peroxidation of lipids, and nitration of protein residues. Papaya leaf extract has shown a significant neuroprotective effect against aluminum-induced cognitive impairment and associated oxidative damage in an animal model [97]. Taking into account the presence of various alkaloids like carpaine, pseudocarpaine, dehydro-carpaine, and phenolic compounds, papaya leaves have been used as antispasmodic, analgesic, and antibacterial. Moreover, the boiled papaya leaves along with some other plant parts such as stem bark and leaves of few medicinal plants were recommended for the treatment of arthritis and rheumatism like inflammation as well as for wound healing too [98]. Studies conducted by various researchers on neuroprotective, anti-inflammatory, and antibacterial effects is summarized in Table 6.

## 4. Papaya Leaf-Based Products

Though papaya leaves are a storehouse of many pharmaceutical properties, they have not been fully utilized up to date on a commercial scale for the production of different formulates. However, a few research studies have documented the development of leaf extract-based products of papaya for efficient utilization of its leaves.

### 4.1. Herbal Juice Beverages

For the preparation of aqueous extract of papaya leaves, the latter are needed to be collected excluding the sap and stalk for the extract preparation. Papaya leaf extract is prepared by chopping or crushing papaya leaves after proper washing, followed by water boiling in a saucepan allowing it to simmer till half-volume reduction and then by straining with a muslin cloth and filling in a glass container [103, 104]. Papaya leaf extract was made by putting papaya leaf powder in a Soxhlet extractor with ethanol and ethyl acetate (95%) for seventy-two hours, and after extraction, the leaf extract was filtered and concentrated through a rotary evaporator [105, 106]. Some researchers crushed the papaya leaves and completed the extraction in a round bottom glass flask at 80°C with water addition at three different times [105]. All the latter washings were blended and distilled under vacuum. The obtained syrup was dehydrated in a vacuum oven to obtain approximately papaya leaf extract. The juice was extracted by the cold juicing method, which has been reported to release bioactive components with more cytotoxic effects in comparison to other aqueous and ethanol isolates. In particular, leaves were washed with mineral water before slicing, and a grinder was used to extract papaya leaf juice. Approximately one kilogram of papaya leaves was blended in mineral water of 250 mL volume in the ratio of 1 : 0.25, and further, the juice was separated from papaya leaf waste through filtering to obtain a clear extract [72, 77].

The bitter taste of leaf extract makes its processed products undesirable for the consumers. A few studies reported the preparation of leaf extract and its blending with fruit pulps for achieving different processed products like jam and beverages, as an alternative for exploiting the leaf extract nutraceutical properties. Previous research revealed that the supplementation of a ready-to-serve beverage based on papaya leaf juice incorporated in guava for the treatment of dengue fever is very safe, as it also induced fast increment in the platelets count and improved immunity in its consumers.

Ready-to-serve beverages were prepared by blending papaya leaf and guava juice aiming to get early recovery of viral disease [10]. Several beverages produced by mixing papaya leaf juice in the banana pulp, pineapple, sweet orange, and pomegranate juice with the addition of papaya leaf extract were prepared to raise the nutritional composition of all the beverages [103]. Standardized formulation for preparation of mango-papaya leaf extract mixture-based nectar has been reported. Prior to beverage preparation, the aforementioned researchers extracted the juice by two hot pressing method, by heating the crushed papaya leaves for 10 minutes, and next passing them through the screw type juice extractor to extract the juice. Further, the leaf extract with 20% water was blended with mango pulp in the ratio of 70 : 30, so as to prepare the final good-flavoured nectar [98].

### 4.2. Herbal Green Tea

Papaya leaves are currently being used for the preparation of green tea in very few countries, thanks to their potent medicinal properties [10, 106]. Enzymes present in papaya leaf green tea have powerful anticancer activities against different types of tumors [36]. For drying, fresh leaves are washed and kept at ambient temperature for at least two hours, and then, they are cut into small pieces and weighed. For the complete removal of free moisture, fresh leaves are placed in mechanical cabinet dehydrator at a temperature of 60°C for few hours, followed by oven drying at 110°C just for a few minutes. After drying, leaves are crushed and packed in bags to store them in a cool and dry place [107].

### 4.3. Herbal Juice Powder and Capsules

Processing plant juices into powder form is a new method to increase the shelf life of the product at room temperature as well as for easy transportation of the reduced volume commodity. Spray drying is a technique applied in the processing industry to obtain fruit juice powder efficiently under controlled conditions. In order to keep these points in mind, researchers have performed spray drying of papaya leaf extract with a certain carrier material to preserve its internal constituents. Before spray drying, the papaya leaf juice is filtered twice to avoid blocking of the spray dryer atomizer. Maltodextrin at a concentration of 8.0-10% *w*/*v* as a carrier material is used to lower down the hygroscopicity of the prepared papaya leaf dried powder. After mixing with maltodextrin, the concentrated papaya leaf juice is conveyed into the spray drying machine with a feed flow rate of 350 mL/h and inside temperature of 130°C*. Papaya* leaf powder is stored under normal room temperature conditions [108].

Freeze-drying or lyophilization is another drying technique for the preparation of good quality juice powders. It is a preferred method for drying of food having compounds that are heat sensitive and vulnerable to oxidation, taking into account that it operates at low temperatures under vacuum [109]. The ice sublimation during freeze-drying protects the main structure and the dried product shape, with minimal volume reduction, higher nutrient, and phytochemical retention due to low temperature. This technique has been successfully applied to diverse biological materials, and in this respect, researchers performed freeze-drying of the papaya leaves after proper cleaning with a veggie wash to scavenge unwanted soil and wax [110]. Further, they washed with reverse osmosis or mineral water prior to juice extraction in a juicer without water addition. After juice extraction, the residual husk was pressed with a clean cloth. The extracted juice was poured in a presterilized glass container and frozen at a temperature of -50°C prior to lyophilization in the dryer, so as to obtain the freeze-dried powdered form of papaya leaf juice with its maximum bioactive components. The obtained powder can be either stored in vials or encapsulated into capsule form for further applications [59]. Researchers dried papaya leaves before isolating them in a conical glass flask at 80°C three times with triple volume of mineral-free water. They collected all the obtained washings and distilled them under a vacuum of 20-30 TDS. The crude form of the obtained extract coming from the syrup dried in a vacuum oven can be used to form capsules [60].

### 4.4. Papaya Leaf-Based Silver Nanoparticles (AgNPs) as Health-Promoting Ingredient

Nanoparticles are an efficient mode for delivering drug nowadays, though the methods used for their synthesis are energy demanding and need harmful chemicals. Recently, researchers have been focusing on environmentally friendly methods of nanoparticles synthesis. The use of plant parts with valuable medicinal properties, appropriate microorganisms, and enzymes has been found effective as alternatives to antibiotics [111, 112]. Nanoparticle biosynthesis has been done by using strains of microbes, proteins, and other metabolites, plant extracts, and biodegradable products. Green synthesis of nanoparticles by utilizing *C. papaya* leaf extract has shown potent antimicrobial attributes. *C. papaya* silver nanoparticles biosynthesis is performed by adding a silver nitrate solution into *C. papaya* leaf extract, with the silver ions getting reduced due to chemical reaction between aqueous extract of leaf extract and silver nitrate solution. The *C. papaya* leaf silver nanomaterial, 5 to 200 nm sized, tends to penetrate into microbial cells and exhibit bacterial cell lysis. Due to antimicrobial effect of the *C. papaya* leaves, silver nanoparticles become more efficient with this extract, exhibit a great bactericidal efficiency, and could act as an alternative to antibiotic resistance [111]. More details on the synthesis of AgNPs from *C. papaya* leaves are discussed in Table 7.

## 5. Safety Assessment of Papaya Leaves

Various scientific studies revealed a great and selective growth inhibitory effect of different plant parts, whose beneficial components may work through different pathways to generate good biological responses. On the contrary, undesired components or even high concentrations of beneficial components in the same parts can generate toxic or side effects, and to minimize the latter effects, the fractionation and standardization of their doses are needed. Papaya leaves have been utilized for the treatment of viral fevers, various cancers, etc., but despite their great uses, literature research reports regarding their detailed toxicity are not available yet up to date.

Studies on the acute toxic effect of papaya leaf extract at a concentration of 2.0 g/kg of the rat bodyweight have been conducted [28]. The latter authors found that the single oral dose of the papaya leaf extract did not turn out the reason of mortality without any significant transformations in the body weight and change in water and food consumption behavior; on the other hand, hemoglobin, red blood cells, and proteins were greatly raised. However, no deaths and no signs of toxicity were recorded during two weeks of investigation. Scientists performed a chemical analysis of *C. papaya* leaves and found that their extract had a significant amount of carpaine, manghaslin, organic acids, clitorin, nicotiflorin, rutin, and other minor chemical constituents [121]. These phytochemicals do not generate treatment-related change in body weight, food and water consumption habits, haematological parameters, and serum chemistry in treated rats after oral administration of the extract for one month. The dose of papaya leaf extracts up to 2000 mg/kg was considered relatively nontoxic during their studies. Similarly, studies to the evaluate the subchronic toxicity effect of papaya leaf extract in rats with the administration of extract prepared from lyophilized leaves powder in clean water papaya leaf extract at concentrations of 0.00, 0.01, 0.14, and 2.00 g/kg weight of rats for almost three months were conducted [122]. Mortality, food, and water consumption behavior were recorded throughout the experimental duration. Their study revealed that leaf extract given for three months did not cause changes in the water and food intake behavior or the bodyweight of the treated rats and concluded that oral administration of papaya leaf extract for three months did not have any type of toxic effect in the rats [123].

In contradiction to the above-mentioned studies, some evaluated the effect of aqueous extract of papaya on rats and found that some concentrations were toxic in different ways [124]. The lethal concentration of the leaf extract with more than 5 g/kg body weight showed no signs of autonomic in acute studies. However, their results revealed that the aqueous extract of papaya had an adverse effect on liver and reproduction system in the rats. In the subacute study, rats were supplied with extract as much as10-500 mg/kg of body weight for two weeks, and they found no effect on the formed elements of blood or haemoglobin, through an injury to the hepato-biliary system. In adult male rats, a significant reduction in sperm count, sperm viability, and testosterone was also observed, and in addition, female rats also showed fertility problems with increase of maternal mortality [125]. Therefore, it is essential to perform descriptive studies to identify and evaluate the leaf chemical constituents, their concentrations, and toxic side effects for further proper utilizations of papaya plant parts, in order to commercialize the relevant products for nutraceutical purposes.

## 6. Conclusions

Research on papaya leaves has not yet received the attention it deserves throughout the world, despite the fact that the bioactive components found in the aforementioned plant parts should be harnessed for nutritional as well as therapeutic objectives. Indeed, papaya leaves have great potential to treat viral infections, to boost immunity along with antidiabetic, anticancer, anti-inflammatory, and many other disease preventive properties. More research studies are required to corroborate the main mechanisms of action shown by the phytochemicals present in papaya leaves as medicinal agents. In the latter respect, various studies found that papaya leaf extract resulted inhibitory effect on cancer cell growth and helped to reduce blood glucose levels. However, further descriptive clinical research is to be carried out to elucidate the functional properties of papaya plant parts on cancer cells and diabetic patients. The consumption of papaya leaves has been shown to have significant benefits in the recovery from viral illnesses like dengue fever. As new emerging viral infections have emerged as a major concern around the world, and as antiviral medications are currently unavailable to cure such diseases, the search for an alternative strategy for lethal corona virus-like diseases has become an urgent priority in recent years. Therefore, the evaluation of papaya leaf extract can be performed as preclinical trials or case-studies, in order to examine the actual effect of this extract and phytochemical responsible for preventing/curing other viral infections on human health.

It is necessary to do additional research in order to individually isolate phytochemicals, define their structure and medicinal properties, standardize the optimum doses, and investigate their toxicity. Because of their valuable phytochemical composition, papaya leaves have the potential to become a new functional food or nutraceutical for the future generations. However, only a few scientific studies have been conducted to date to formulate different papaya leaf-based products, and the commercial application of these products should be appropriately investigated. The leaves of this horticulture plant that is grown in underdeveloped nations have the potential to become a very attractive source of extremely nutritive and medicinal phytochemicals in the not-too distant future.

## Figures and Tables

**Table 1:** Antioxidant activities of *Carica papaya* L. leaf extract.

Type of extract	Method used	Responsible phytochemicals	References
Methanol	Peroxynitrite scavenging assay	Kaempferol 3-(2G-rhamnosylrutinoside)	[41]
Ethanol, methanol, and water	DPPH, FRAP	Flavanoids	[44]
Methanol	DPPH	Carpaine, kaempferol 3-(2G-glucosylrutinoside), kaempferol 3-(2^″^-rhamnosylgalactoside), 7-rhamnoside, kaempferol 3-rhamnosyl-(1->2)-galactoside-7-rhamnoside, luteolin 7-galactosyl-(1->6)-galactoside, orientin 7-O-rhamnoside, 11-hydroperoxy-12,13-epoxy-9-octadecenoic acid, palmitic amide, and 2-hexaprenyl-6-methoxyphenol	[25]
Methanol	DPPH	—	[45]
n-Hexane, dichloromethane, ethyl acetate, ethanol, methanol, n-butanol, and water	DPPH	Phenolics and flavonoids	[46]
Aqueous	DPPH, ABTS	Polyphenols	[5]
Methanol	Phosphomolybdenum method	Flavonoids	[47]
Aqueous	DPPH, ABTS^+^ assay	Proteins and phenolic groups	[39]

**Table 2:** Medicinal potential of *Carica papaya* L. leaf extract against virus-induced thrombocytopenia.

Treatment	Results	References
Mature *C. papaya* leaf concentrate (0.72 mL/100 g bw of adult Wistar rats) administered for 3 days	(i) Increase in platelet count without toxicity in rats (ii) Increase in platelets by 76.50%, WBC by 30.51%, and RBCs by 9.08%	[57]
Fresh *C. papaya* leaf extract (0.2 mL (2 g)/mouse) for twenty-one days	(i) Increment in the platelet and the RBC count. (ii) The platelet count reached almost a fourfold higher at day 21 (11.3 × 10^5^/*μ*L) and RBC count in the test group increased from 6 × 10^6^/*μ*L to 9 × 10^6^/*μ*L at the end of treatment.	[7]
*C. papaya* extract (150 mL) daily to dengue patient for five days	(i) Increase in no. of in thrombocytes (28 · 103/mL to 138 · 103/mL) and white blood cells BC (3000/mL · 7800/mL) in a dengue adult patient	[52]
Administration of 500 mg papaya leaf extract capsules on daily basis along with supportive medical treatment for five days to patients	(i) Increment in platelet count (ii) Third day onwards platelet count showed significantly positive results in the study group (82.96 ± 16.72) than control (66.45 ± 17.36). This trend of significant difference was the same on the fourth and fifth day of their studies. (iii) Average platelet transfusion requirement in the study group was significantly less than the control group (0.685 units per patient vs. 1.19 units per patient)	[59]
Carpaine extracted from *C. papaya* leaf (2 mg/kg BW of thrombocytopenic Wistar rats) for twenty days	(i) Isolated carpaine from *C. papaya* leaf extract exhibited potent activity in sustaining normal platelet counts without acute toxicity.	[60]
Aqueous extract of *C. papaya* leaves (one spoonful of leaf paste) extract on dengue-infected children for two days	(i) Increase in number of a platelet count of dengue-infected children of age 10 and 14 (ii) After one-day administration, platelet count was 100,000 and within 2 days count reached up to 250,000	[61]
Aqueous extract of *C. papaya* leaves (25 mL) twice a day for two days	(i) Significant increase in the platelet an white blood cell count after 2 days of treatment	[62]
Standardized *C. papaya* leaf aqueous extract (50 and 150 mg/kg BW of Wistar rats) for two weeks	(ii) Oral administration showed a significant increase in thrombocytes (1014.83 cells/mm^3^), DTH response (0.16), and phagocytic index (63.15% increase)	[63]
*C. papaya* leaf extract capsules (290 mg) dose daily twice in thrombocytes postchemotherapy cancer patients for five days	(i) After 5 days, the mean increase in platelet counts from 101.93 × 10^3^/*μ*L to 173.75 × 10^3^/*μ*L	[64]
Administration of papaya leaf extract (1.1 g) to total five hundred patients suffering from thrombocytopenia three times daily for five days	(i) A significant increase in counts of platelets were noticed in the study group.	[65]
Treatment of infected mice with 1000 mg/kg bw of FCPLJ (freeze-dried *C. papaya* L. leaf juice content) for four days	Increase in the number of total white blood cell and neutrophil counts by 1.44-fold.	[66]

**Table 3:** Medicinal potential of *Carica papaya* L. leaf extract against virus-induced thrombocytopenia.

Treatment	Effect on cancerous cells	References
Aqueous isolate of *C. papaya* leaves (1.25–27 mg/mL)	(i) Exhibited an effective anticancer property on cancer cell lines (stomach cancer cell line (AGS), pancreatic cancer cell line (Capan-1), colon cancer cell line (DLD-1), ovarian cancer cell line (Dov-13), lymphoma cell line (karpas), breast cancer cell line (MCF-7) (ii) Suppressed DNA synthesis by inhibiting the incorporation of 3H-thymidine	[70]
Aqueous extract of *C. papaya* leaves (0.625–20 mg/mL)	(i) Inhibition of proliferative responses of haematopoietic cell lines and solid tumour cell lines (ii) Increase in the expression of immune modulatory genes	[36]
Brewed leaf juice (20 mg/mL)	(i) Effective antiproliferative activity against cancerous cells of the prostate (ii) Suppression of SCC25 cells growth in a dose-dependent manner (iii) Survivability of 20% SCC25 cells, and 70% cancer-free human keratinocyte HaCaT cells remained viable with a dose of 20 mg/mL	[37]
Aqueous *C. papaya* leaf extract (659.63 *μ*g/mL)	(i) Antiproliferative and apoptotic induced effect of papaya leaf inhibits the proliferation of human breast cancer cell (ii) Leaf extract exhibited apoptosis of MCF-7 cell line (22.54%)	[76]
Papaya leaf juice and its various extracts (0.25–0.1 mg/mL)	(i) Effective antiproliferative activity against cancerous cells of the prostate (ii) Potent growth inhibitory and cytotoxic activities on all prostate cells except the normal (RWPE-1 and WPMY-1) cells (iii) Medium polar fraction inhibited migration and adhesion of metastatic PC-3 cells	[77]
*C. papaya* leaf juice (0.01-1 mg/mL) in prostate epithelial cancer cells, benign tumor, and human prostate cancer cells	(i) Decrease the cancer cell proliferation (ii) Arrest the S phase cell cycle (iii) Induced apoptosis in prostate cancer cells	[7]
Silver nanoparticles (AgNPs) with papaya leaf extract (0.5, 1, 2.5, and 5 *μ*g/mL) at 24 h and 48 h on human prostate carcinoma DU145 cells.	Reduction in cell proliferation and subsequent apoptosis of human prostate carcinoma DU145 cells.	[78]

**Table 4:** Immunomodulatory potential of papaya leaf extract.

Treatment	Results	References
Oral administration of SCPLE (150 mg/kg) in thrombocytopenic rats	Significant (*p* < 0.01) increase in thrombocytes (1014.83 × 10^3^ cells/mm^3^), DTH response (0.16 ± 0.004), and phagocytic index	[63]
Administration of *C. papaya* and methanol (MeOH) extracts on mice for 3 weeks.	Proinflammatory cytokines (IL-10, IL-12, IL-1*β*, IL-6, and TGF-*β*1) were decreased.	[80]
Aqueous-extracted CP leaf fraction on the growth of various tumor cell lines	Production of IL-2 and IL-4 was reduced. IL12p40, IL-12p70, IFN-, and TNF- were enhanced without growth inhibition	[36]

**Table 5:** *In vivo* studies on the medicinal potential of *Carica papaya* L. leaf extract against diabetes.

Treatment	Results	References
The ethanolic aqueous extract of *C. papaya* (100 mg/kg) with water given to streptozotocin-induced diabetics for five days.	(i) Reduced glucose levels in the blood at the end of the fifth day of treatment. (ii) A great regeneration of the tissues of the liver. (iii) The tissues of kidney indicated a great recovery in the cuboidal tissue	[89]
The aqueous extract of *C. papaya* (0.75 g and 1.5 g/100 mL) for one month	(i) Delay in attaining the maximum plasma concentrations of amiodarone, extract and amiodarone increased the drug bioavailability (ii) Significant elevation in serum glucose levels (434.0 mg/dL) in comparison to the untreated rats (iii) Significant decrease in blood glucose levels up to 306.00 10.2 mg/dL	[90]
Aqueous extract of *C. papaya* leaf extract (400 mg/kg BW of diabetic albino rats) for twenty-one days	(i) Significant reduction in blood glucose level and serum lipid profile levels due to antihyperglycemic and hypolipidemic properties. (ii) Leaf extract showed 38.19 per cent reduction in the blood glucose level after completion of treatment	[91]
Ethanolic extract of *C. papaya* leaves (250-500 mg/kg BW of alloxan-induced diabetic rats) for twenty-one days	(i) Significant reduction in glucose level (123.50 mg/dL), total cholesterol, triglyceride (1.24 mg/dL), and serum urea (12.35 mg/dL). (ii) Significant increase in HDL cholesterol and total protein level (66.51 g/dL). (iii) Significant decrease in LDL cholesterol, creatinine, alanine aminotransferase and aspartate aminotransferase.	[92]
Ethanolic leaf extracts of *C. papaya*, i.e., 50, 150 and 300 mg/kg BW of diabetes-induced mice.	(i) Good effects on plasma insulin, cholesterol, triglyceride, and HDL cholesterol levels (ii) Hypoglycemic effect in diabetic rats after taking various doses of *C. papaya* extract	[87]
Aqueous extract of *C. papaya* leaf (120 mg/kg BW of albino rats) for eighteen days	(i) Significantly reduced glucose level from 275.00 to 85 mg/dL, total cholesterol from 117.70 to 98.50 mg/dL, total glycerides from 107.10 to 97.21 mg/dL, and LDL from 49.44 to 44.01 mg/dL	[93]
The administration at a dose of 1000 mg/kg body weight of papaya leaf ethanol extract in diabetic Wistar mice.	Reduce blood glucose levels in diabetic Wistar mice	[94]

**Table 6:** Medicinal potential of *Carica papaya* L. leaf extract as neuroprotective, anti-inflammatory, and antibacterial herbal medicine.

Treatment	Results	References
Ethanolic extract of *C. papaya* leaves (25–200 mg/kg), carrageenan-induced paw oedema, cotton pellet granuloma, and formaldehyde-induced arthritis rats for ten days	(i) Reduction in inflammation and in carrageenan-induced paw edema, granuloma (cotton pellet induced) in arthritic mice (ii) Significant decrease in the amount of granuloma from 0.58 to 0.22 g	[99]
Aqueous *C. papaya* extract (0.625–2.5 mg/mL) in infected human beings	(i) Increment in protein content and also increased production of antibodies against ovalbumin (ii) Exhibited anti-inflammatory and antimicrobial activities at higher doses (iii) Leaf extract exhibited a decrease in gram-positive and gram-negative bacterial count and proliferation rate.	[100]
Alcoholic *C. papaya* leaf extract 200-400 mg/kg BW of male Wistar rats for forty-two days	(i) Neuroprotective effect of *C. papaya* leaves on an animal model (ii) Denoted a significant elevation in the level of acetylcholine, SOD, glutathione, and catalase and great reduction in total proteins	[101]
Methanol and aqueous extracts of *C. papaya* leaves (25 mg/mL–100 mg/mL).	Inhibits the growth of *Staphylococcus aureus, Escherichia coli*, and *Candida albicans*	[19, 102]

**Table 7:** AgNPs synthesis using *C. Papaya* leaf with well-defined applications, characterization techniques, particle characteristics, and operating conditions.

Applications	Operating conditions	Characterization techniques used	Particle characteristics	Reference
(i) AgNPs exhibited inhibitory effect against both gram-positive and negative bacterial species	AgNO_3_ (1 mM); extract: AgNO_3_ (1 : 4), heated at 60°C for 5–10 min and incubated on sand bath for 30 min	UV-vis, FTIR, SEM-EDX, TEM, XRD	Size: 50-200 nm; shape: spherical	[112]
(i) Antibacterial activity against human pathogens such as *Bacillus subtilis*, *Enterococcus faecalis*, *Escherichia coli*, *Vibrio cholerae*, *Klebsiella pneumoniae*, and *Proteus mirabilis* (ii) Cytotoxic effects against human breast carcinoma cell line (MCF 7)	AgNO_3_ (2 mM); kept at 37°C in dark condition for 72 h	HR-TEM, UV-vis, FTIR, XRD	Size: 6-18 nm shape: face-centered cubic crystalline (fcc)	[113]
(i) NPs exhibited excellent antibacterial activity against *P. aeruginosa* and *E. coli*. (ii) Exhibited binding activity against dengue type 2 virus NS1	AgNO_3_ (1 mM); aqueous leaf extract: AgNO_3_ (1 : 9); maintained at 27°C/24 h	FTIR, AFM, XRD	Size: 7–32 nm shape:	[114]
(i) Showed antibacterial activity against *E. coli* and *B. Cereus*	AgNO_3_ solution	UV-vis, FESEM, FTIR, EDX	Size: 13-69 nm shape: spherical	[115]
(i) Showed exhibited inhibitory against both gram-positive and negative bacterial species	AgNO3 (0.01 M); aqueous leaf extract: AgNO3 (1 : 4), heated on a sand bath at 70°C for 20 min	UV-vis, SEM	Size: 5 to 50 nm shape: spherical	[116]
(ii) Antimicrobial activity against *Pseudomonas aeruginosa*, *Escherichia coli*, *Bacillus subtilis*, and *Staphylococcus aureus*	1% silver nitrate (AgNO_3_); maintained at 40°C temperature for 24 h.	UV-Vis	Size: 250 nm shape: ND	[117]
(i) Antioxidant	AgNO_3_ (1 mM); extract: AgNO_3_ (1 : 9); mixture was boiled at 45°C for 30 min	HE-TEM, XRD, FE-SEM, EDX	Size: 10 nm shape: spherical	[118]
(i) Blue CP and yellow 3RS dyes degradation (ii) Antibacterial activity against gram negative (*E. coli*) and gram positive (*S. aureus*)	AgNO_3_ (0.002 M); incubated for 24 h at 37°C	TEM, EDS, UV-vis	Size: 10-70 nm Shape: spherical	[119]
(i) Antibacterial activity against human pathogens (*Escherichia coli* and *Staphylococcus aureus*)	AgNO_3_ (1 mM)	UV–vis, FTIR, XRD, SEM, TEM	Size: 16 nm to 18 nm shape: spherical	[120]
(i) Better efficacy against cancer cells and was also relatively less toxic to normal cells	AgNO_3_ (1 mM); 200 *μ*L extract: 10 mL AgNO_3_); solution was incubated in water bath at 50°C	TEM, STM, SEM, EDS, XRD, FTIR	Size: 10 to 20 nm shape: spherical	[7]
(i) Exhibited an excellent antibacterial activity against gram-positive and gram-negative pathogenic bacterial strains like (*Klebsiella pneumoniae*, *Staphylococcus aureus*, *Escherichia coli*, and *Yersinia enterocolitis*)	AgNO_3_ (1 mM); extract: AgNO_3_ (1 : 9); incubated for 24 h at 37°C	UV–vis, FTIR, SEM, EDX, DLS	Size: 80 nm shape: spherical	[111]
(i) Possessed potential antibacterial activity against *Escherichia coli*	AgNO_3_ (1 mM); kept at 37°C for 3 hours	UV-vis, TEM, FTIR	Size: 13-17 nm shape: spherical	[121]
